# Biomedical research ethics in Cameroon: a survey to assess training needs of medical residents and students

**DOI:** 10.1186/s12909-018-1431-8

**Published:** 2019-01-03

**Authors:** Jerome Ateudjieu, Samia Hurst, Martin Ndinakie Yakum, Godfrey B. Tangwa

**Affiliations:** 1 M.A. SANTE (Meilleur Accès Aux Soins de Santé), Yaoundé, Cameroon; 2 Geneva University Medical School, Institute for Biomedical Ethics, Geneva, Switzerland; 3 Department of Phylosophy, University of Yaounde 1, Yaounde, Cameroon

**Keywords:** Research, Bioethics, Ethics, Training-needs

## Abstract

**Background:**

Training curricula in research ethics for potential and future researchers should be implemented and constantly updated. This requires data regarding training needs.

**Methods:**

We conducted a cross-sectional survey on residents, fifth and sixth-year medical students registered in the 2006–2007 academic year at the Faculty of Medicine and Biomedical Sciences (FMBS) of the University of Yaounde 1, Cameroon.

**Results:**

Two-fifths (40.4%) of respondents (response rate 70.9%) reported training in research ethics. Less than half were aware of specific regulatory texts relevant to research ethics. Among those who reported conducting a research project 66.7% declared having obtained informed consent from participants and 32.9% having submitted their protocol to an Ethics Review Committee. Participants identified training priorities in research ethics and responsibilities of key actors in the protection of research participants.

**Conclusion:**

There is a need to improve the training and attitude of medical students and residents in order to prepare them to respect and protect research participants.

**Electronic supplementary material:**

The online version of this article (10.1186/s12909-018-1431-8) contains supplementary material, which is available to authorized users.

## Background

Researchers have a key role in the protection of biomedical research participants [1, 2]. However, in ethical terms, this role is frequently neglected and can be threatened by the conflict of interest inherent in any research [3]. It has been documented that, for reasons which include financial and/or career stakes, exaggerated enthusiasm, influences from various sources and even ignorance, researchers have – intentionally or not – designed and/or implemented research projects which neglect the lives, health, privacy and dignity of research participants [4–6]. This situation has been reported in both developed and developing countries [7–11].

It is reasonable to expect a positive impact on the research quality and on the protection of research participants if residents’ and medical students’ training curricula in biomedical research ethics are adapted to what they would perceive, and what would be documented as, their training needs. This is supported by the following facts: in many African countries, the majority of researchers and members of Research Ethics Committees (RECs) are physicians [12–15]; training in biomedical ethics has been perceived by medical students in some developed countries as having a positive influence on their attitudes and practices concerning ethical issues [16–18]; and introducing biomedical ethics in student curricula has been identified as a key strategy that can strengthen awareness of biomedical ethics and research participant protection [19].

We therefore conducted a survey to evaluate the perceived and documented training needs of residents and medical students preparing to conduct their academic research projects. One purpose was to point out elements which should be incorporated into residents’ and medical students’ research ethics curricula. The present study aimed to answer the following questions: what was the coverage rate of research ethics training in the Faculty of Medicine and Biomedical Sciences (FMBS)? What were the training sources? To what degree were residents and medical students aware of research ethics regulations and ethical principles? What were their responsibilities in the protection of research participants? What were their attitudes regarding research participants’ protection while implementing their research projects? What did they perceive as important and as training priorities in biomedical ethics? Conducting a survey was an adequate, if not the only, research method to find reliable and valid information on these questions. To the best of our knowledge, no study had previously been conducted in any Cameroonian or sub-Saharan African university to provide such information. The FMBS was selected because, uniquely in Cameroon, it simultaneously trains medical students and residents who have either conducted, are conducting or will soon conduct biomedical research projects. In order to contribute to improving biomedical research ethics standards and the protection of research participants, we conducted this study from January to November 2007, with the objective of assessing the biomedical research ethics training needs of residents, as well as fifth and sixth-year medical students, registered in the FMBS.

The FMBS is part of the University of Yaoundé 1. It was created in 1969 and currently provides training for medical doctors over seven academic years and two to four academic-year residencies in nine specialties. Medical students receive basic biomedical training in their first three academic years, then practical clinical and public health training from the fourth to the sixth academic years.

Residents registered at the FMBS are medical doctors with at least two years’ field experience. They have been trained for two to four academic years, depending on the speciality. They receive both basic and applied training through lectures, seminars and clinical internship. As part of their training programme, each resident must successfully design, implement and defend a biomedical research project in front of a jury. Each resident’s research project is supervised by a team of qualified professors.

## Methods

We designed a survey to evaluate the perceived and documented training needs of residents and medical students preparing to conduct their academic research projects. The questionnaire (see S1) was inspired by a questionnaire used for a similar study [20]. It was circulated within the research team for inputs. Pre-testing was conducted in a group consisting of two medical students and three residents from the FMBS. The resulting revised questionnaire was validated with regard to its reliability, validity and timing (30 min). Data were collected using a semi-structured questionnaire (see Additional file 1) targeting respondents training in bioethics, awareness of regulations and bioethics principles, and perception and attitude of respondents in research participants’ protection**.** The reliability and validity of the questionnaire were simply appreciated after the pre-test by bioethics experts who were members of the research team. Before its implementation the protocol was approved by the Cameroon National Ethics Review Committee.

This study was a descriptive cross-sectional study. It targeted all residents, fifth and sixth-year medical students registered in the FMBS for the academic years 2006–2007. With the permission of the FMBS’ academic authority, we contacted class representatives of medical students and residents to obtain timetables and lists of potential participants. In accordance with these lists, we targeted 364 students, of whom 265 were reached by the surveyor and invited to participate in the study. They were provided with full and easily understandable information about the study, as well as required explanations. Residents’ and medical students’ representatives were interviewed regarding the contents of their training curricula. The majority were contacted during a break before or after their classes or internship activities. In order to avoid disrupting their scheduled activities, we gave them the questionnaire to be self-administered at their preferred time and returned with a signed informed consent sheet. Respondents were asked to express how they viewed the responsibilities of key actors in the protection of research participants. Their responses were scored as follows: 3 for ‘very important’, 2 for ‘moderately important’, 1 for ‘less important’ and 0 for ‘not important’.

Respondents were asked to express their views on the importance and priority of proposed training topics. Their responses were scored as follows: 3 for ‘very important’, 2 for ‘moderately important’, 1 for ‘less important’ and 0 for ‘not important’. The perceptions of respondents regarding proposed training topic priorities were also scored: 3 for first-degree priority, 2 for second-degree priority, 1 for third-degree priority.

Data were coded and entered in Epi info software version 3.2 and analysed using the same software, and Excel version 5.0. Scores were attributed to respondents’ perceptions of the importance and priorities of proposed training needs and the responsibilities of key research actors regarding the protection of research participants. Data entry was controlled using a code book, pre-setting values labels, double entry by two secretaries and keeping completed questionnaires and study diaries for later control. The analysis was done by calculating and comparing frequencies, ranking and means. The independent Chi-2 test compared proportions. We accepted a significance level of 0.05.

## Results

### Respondents

Out of 364 potential participants, including 176 medical student*s* and 188 residents, 265 were contacted and received the questionnaire (coverage rate of 72.8%) and 188 responded, making a response rate of 70.9%. Those who were not reached by the survey were mostly those conducting their research in hard-to-reach areas with poor communication network. No data was collected on potential participants not reached. Ninety-one (48.4%) respondents were medical students, including 41 fifth-year medical students and 50 sixth-year medical students. Ninety-seven (51.6%) respondents were residents, including six residents in Pathology, eight in Anaesthesiology, 17 in Clinical Biology, 10 in Surgery, 11 in Obstetrics and Gynaecology, eight in Medical Radiology, 11 in Internal Medicine, 15 in Paediatrics and 11 in Public Health. The response rate among residents (79.5%) was significantly superior to that of medical students (63.6%) (*p* < 0.001). Ninety-three (49.5%) respondents were women and 95 (50.5%) were men.

### Training received by respondents

Seventy-six respondents (40.4%) reported having received training in research ethics. Table 1 shows the distribution of those who reported having been trained by class and by specialty. The proportion of medical students reporting training (53.9%) was significantly higher than that of residents (27.8%) (*p* < 0.0001). However, 100% of Public Health residents reported training. Overall, the training was received either in workshops (33.7%) or academic courses (66.3%).

**Table 1:** Distribution in classes and specialities of respondents regarding training received in research ethics

Classes and specialities	Number of respondents	Reported having received training in research ethics	
		Number	Proportion (%)
6th year medical students	50	26	52.0
5th year medical students	41	23	56.1
Anaesthesiology	8	1	12.5
Clinical Biology	17	1	5.9
Internal Medicine	11	1	9.1
Medical Radiology	8	4	50.0
Obstetric and genecology	11	3	27.3
Paediatric	15	4	26.7
Pathology	6	1	16.7
Public Health	11	11	100.0
Surgery	10	1	10.0
Total	188	76	40.4

### Biomedical research ethics in training curricula

In the FMBS, research ethics training has been part of the training curriculum of fifth-year medical students since 2006. Among residents, only Public Health residents have had this course in their training curriculum since 2005. In terms of content, the training provided to fifth-year medical students consisted of an introductory course, historical overview of research ethics, fundamental principles of ethics, informed consent, research with vulnerable populations, ethical issues of research conducted in developing countries, the role of RECs and ethical considerations in designing research protocols. The Public Health resident training programme was reported to have the same training content plus training in international research regulations. The reported contents of workshops were much diversified. The mean duration of training was reported as 18.9 ± 14 h. Research ethics training was part of the examination subjects for Public Health residents but not for medical students.

### Satisfaction of respondents regarding received training

As regards satisfaction with the training they received, 14.3% reported being very satisfied, 44.9% moderately satisfied, 30.6% less satisfied and 10.2% not satisfied. Additionally, 99.5% of respondents wished to receive more training in biomedical ethics, while 0.5% did not feel this need.

### Respondents’ awareness of regulatory and biomedical ethics principles

Tables 2 and 3 indicate respondents’ awareness regarding, respectively, regulatory texts and principles guiding the protection of human participants in research projects. Among key research ethical international regulations, the Helsinki Declaration was most frequently cited. Indeed, 30.2 and 12.6% were aware of its existence and content, respectively.

**Table 2:** Awareness of respondents on the existence and content of key international and Cameroon regulatory texts guiding the protection of human research participants

Regulatory texts	Number of respondents	Proportions of respondents		
		Not aware of existence	Aware of existence only	Aware of existence and content
		% (n)	% (n)	% (n)
Specific to research ethics				
Helsinki Declaration	182	57.1 (104)	30.2 (55)	12.6 (23)
Nuremberg Code	180	66.7 (120)	24.4 (44)	8.9 (16)
CIOMS International Ethical Guidelines for Biomedical Research Involving Human Subjects	181	74.6 (135)	15.5 (28)	9.9 (18)
The WMA Declaration on Ethical Considerations Regarding Health Database	182	81.9 (149)	13.7 (25)	4.4 (8)
International Guidelines for Ethical review of epidemiological studies	182	83.0 (151)	12,6 (23)	4.4 (8)
WHO Operational Guidelines for Ethics Committees that Review Biomedical Research	180	84.4 (152)	11.1 (20)	4.4 (8)
The Belmont Rapport	181	89.0 (161)	6.6 (12)	4,4 (8)
ICH-GCP	179	89.4 (160)	6.1 (11)	4.5 (8)
Non-specific to research ethics				
The Hippocratic Oath	183	4.9 (9)	26.8 (49)	68.3 (125)
Universal Human rights declaration	181	9.4 (17)	50.3 (91)	40.3 (73)
Cameroon code of medical Ethics	183	20.8 (38)	53.0 (97)	26.2 (48)
Ministry of Public Health Order Creating and Organizing an Ethical Review Committee in Cameroon	181	54.1 (98)	36.5 (66)	9.4 (17)

**Table 3:** Respondents’ awareness of the existence, significance and capacity to implement key principles guiding the protection of human research subjects

Key principles	Number of respondents	Proportion of respondents reporting			
		No awareness of existence	Awareness of existence	Knowledge of the significance	Ability to implement
		% (n)	% (n)	% (n)	% (n)
Scientific validity	181	34.3 (32)	35.4 (64)	13.3 (24)	17.1 (31)
Independent review	180	23.3 (42)	34.4 (62)	15.6 (28)	26.7 (48)
Respecting research participant	178	25.3 (45)	25.3 (45)	13.5 (24)	36.0 (64)
Favourable risk benefit ratio	181	35.9 (65)	23.2 (42)	21.0 (38)	19.9 (36)
Coverage of damages	179	43.0 (77)	27.4 (49)	16.2 (29)	13.4 (24)
Fair selection of study population	182	45.1 (82)	22.0 (40)	13.2 (24)	19.7 (36)
Social value	183	50.8 (93)	26.2 (48)	10.9 (20)	12.0 (22)
Collaborative partnership	180	67.8 (122)	14.4 (26)	8.3 (15)	9.4 (17)
Informed consent	179	17.9 (132)	19.0 (34)	17.9 (32)	40.8 (73)

### Informed consent and independent review

Seventy-two of 152 respondents (47.4%) reported having been involved in a biomedical research project as principal investigator or co-investigator. Fifty-five (76.4%) were residents and 17 (23.6%) medical students. Forty-six (66.7%) of the protocols were reported to have been implemented with the consent of adequately informed participants. The proportion of those who reported training (48.50%) was similar to the proportion not trained in research ethics evaluation (51.50%). Only 32.9% of their research projects were implemented after submission to an independent REC for review. Among those who received training in research ethics evaluation, 45.8 sought ethical approval before implementing their research protocols meanwhile only 12.1% sought ethical approval before implementing among those with no such training.

### Perception of respondents on the responsibility of key actors

This perception was expressed through scores. Mean values for scores attributed by respondents are presented in Table 4. Ranking of scores per agent indicates that investigators, sponsors and RECs were perceived to have much more important roles in the protection of research participants compared with research participants, physicians or the community.

**Table 4:** Respondents’ perception on the degree of importance of key actors’ responsibilities, regarding the protection of research participants

N°	Actors	Means of scores
1	RECs	2.33
2	investigators	2.32
3	sponsors	2.21
4	physician in charge of research participant care	1.17
5	research participants	1.09
6	community	0.68

### Perception of importance and priority of proposed training topics

Of 25 proposed training topics, regulation of research involving human beings at national level (90.4%) and basic ethical principles (77.3%) were perceived by more than 75.0% of respondents as being very important. Moreover, 14 (56.0%), 2 (8.0%) and 1 (4.0) of training topics were, respectively, perceived by more than 50.0%, more than 25.0% and less than 25% of respondents as being very important.

Table 5 presents the ranking of priorities of proposed training topics by sum of scores attributed by respondents according to their perception. Participants identified and ranked their training priorities.

**Table 5:** Ranking of priorities of proposed training topics by sum of scores attributed by respondents according to their perception

Rank	Training topic	Sum of scores
1	Basic ethical principles	238
2	Regulation of research involving human beings in international context	170
3	Ethical issues in research involving vulnerable populations	76
4	Regulation of research involving human beings in Cameroon	74
5	The process of informed consent	62
6	Scientific validity of a research project	52
7	Respect for research participants	42
8	Assessing the risk/benefit ratio	40
9	Ethical issues in clinical trials and other interventional studies	34
10	Ethical issues in research on records and personal data	20
11	Harm monitoring and compensation for damages	19
12	The notion of conflict of interest	19
13	Incentives, undue incentives and coercion	17
14	Social value of research involving human beings	15
15	Relevance of research to local health needs	13
16	Ethical issues in qualitative research	13
17	Role and responsibility of researchers in the protection of research participants	11
18	Role of REC: authority, mandate and responsibilities	10
19	Publication and authorship issues	5
20	Role and responsibility of the sponsor in the protection of research participants	4
21	Ethical issues in multicentre studies	3
22	Ethical issues in research on stored biological samples	2
23	Process of obtaining community permission for medical research	0
24	Ensuring appropriate and fair selection of research participants	0
25	Ethical issues in externally sponsored research	0

## Discussion

The purpose of this study was to identify the training needs in research ethics for residents and fifth and sixth-year medical students registered in the FMBS and preparing to conduct their academic research projects. About two-fifths of respondents reported having received training in research ethics. Few of them were aware of ethical principles and the contents of the existing national and international regulations in biomedical research. Their attitudes were illustrative of the need for training, on account of the fact that only 66.7 and 32.9% reported having implemented their research projects after having obtained, respectively, the informed consent of participants and the approval of a competent research ethics committee. However, the great majority identified investigators, sponsors and RECs as playing the principal role in the protection of research participants, and thus they rightly perceived their responsibility when implementing research projects. From a list of proposed training topics, they identified and ranked their perceived training needs and priorities.

### Training received by respondents

Despite the fact that all residents and medical students were expected to conduct a research project as a requirement of their training programme, only 40.4% of respondents reported any training in research ethics. This low percentage of trainees reflects the situation in the FMBS, where only 1 of 9 of the residents’ specialities (Public Health) has biomedical ethics training as part of its curriculum. It is also surprising that only 53.9% of medical students reported having received training, despite the fact that it was scheduled and implemented as part of their curriculum. This low rate could be explained by the fact that the courses were not part of examination subjects and there was no negative sanction for those who missed the training.

The low percentage of participants reporting training in research ethics and awareness of key issues explored in this study suggests that biomedical research ethics is afforded low priority by academic authorities, medical students and residents within the medical curriculum. In a context where clinical research is increasingly globalised and where African doctors are likely to be confronted with research ethics issues, both in studies they initiate and as collaborators in international studies, these results should give us pause for reflection. Basic training in research ethics for all medical researchers is essential for the protection of human subjects involved in biomedical research.

From the actual results, it appears that urgent actions should aim firstly to sensitise previous, current and future medical students and residents, who would or would not have benefited from biomedical ethics training in their curriculum, by providing – for example – online courses and workshops. The second objective is to plead for better consideration of biomedical research ethics in the medical curriculum on the part of academic authorities.

### Content of training received by residents

The content of training provided to medical students and Public Health residents matches respondents’ training priorities and covers what are highlighted in key international ethical guidelines as elements contributing to improved protection of research participants [1]; even if outcome of training does not only depend on its content, but also on other aspects not assessed in this study, like the training methodology, the competency of trainers, the ability and motivation of trainees to develop competency from acquired training and whether it responds adequately to local cultural realities and research priorities.

### Awareness of respondents regarding regulatory and biomedical ethics principles

The tenth principle of the 2008 Helsinki Declaration states: ‘Physicians should consider the ethical, legal and regulatory norms and standards for research involving human subjects in their own countries as well as applicable international norms and standards^1^. The present study suggests that in Cameroon, the majority of medical students and residents at the end of their training period are not aware of the existence and content of national and international regulation regarding research ethics. This was indeed the case as regards the Helsinki Declaration and the only national regulation on research participants. As is often the case, the existence of legislation is only the first step. The essential next step is to apply it and make it known. Participants’ awareness of regulations does not guarantee that they will respect ethical principles. It does, however, show that these texts are available to respondents, to be consulted as guidelines on which research participants’ protection should be based. Furthermore, being able to apply ethical principles also implies awareness of their meaning and the ability to take them into account when designing, evaluating or implementing a research protocol.

### Attitude of respondents towards respect for certain ethical obligations

The fact that respondents recognised not having sought research participants’ consent or ethical approval before implementing their research project raises questions not only regarding their attitudes, but also the ethical review process in the FMBS and in the country, as well as the capacity and willingness of respondents’ research supervisors to protect research participants. There is not yet a single law covering research involving human beings in Cameroon. The protection of research participants is, therefore, based on international regulations and local laws, which apply to different sectors of health and the protection of human rights. An ethical committee was created in 1987 by the Cameroon Ministry of Health, although the order guiding its function does not explain its geographical area of competency [21]. It does, however, state that the submission of a research protocol to the ethics committee is an obligation, and includes the informed consent process as part of protocols to be submitted for ethical clearance. To minimise potential risks, action should be taken to improve the national regulatory environment and to make students, supervisors of their theses and FMBS authorities aware of the importance of independent review of biomedical research protocols. Furthermore, since most medical students and residents will, during their professional lives, very likely endorse responsibilities for research, clinical care for research participants, participation in health policy decisions or research ethics committee review research ethics evaluation should constitute an essential part of their culture and core skills [21–23].

### Perception of responsibilities for research participants’ protection

Identifying sponsors, researchers and REC members as prime movers in the protection of research participants as stated in the majority of regulations, even if most were not aware of it, points to the fact that these regulations are based on moral principles that can be applied generally and thus universally [24]. This can be a reason for hope. The same consideration can explain the fact that their choices of important training priorities (see Table 5) was largely similar to the perceived training needs of RECs’ members at national and African level [20]. Despite this similarity in training priorities, these results in the other different and important topics explored are contrasted with the situation in its African context [20]. Their top five perceived training priorities were relevant and constituted the minimum requirements for them to play key roles in the protection of research participants.

### What is expected if identified needs are overcome?

Even if training does not always prove beneficial, making it accessible to all residents and medical students can significantly improve general awareness and competence in dealing with ethical issues in the position of researcher, Ethics Review Committee member, research participant or decision-maker. The fact that in this study the proportion of respondents who sought ethical approval before implementation of research protocols was significantly higher among those reporting training reaffirms its importance. In a previous study, participants recognised that professional attitudes and values are an appropriate focus for medical education. A further study revealed that the clinical faculty’s evaluation of professional judgement during patient care was enhanced by training [25].

### Limitations

This study was limited by some unavoidable factors. Firstly, it targeted all residents, fifth and sixth-year medical students registered in the FMBS in the 2006–2007 academic year, but only 72.8% of this population was reached. This limitation was, however, unlikely to cause bias, because it was not linked to any training needs in research ethics. Secondly, 70.94% of those who received the questionnaire returned it and this response rate was significantly higher among residents than medical students. We did not collect information to ascertain why some did not respond and why more residents than medical students responded. This information could have been useful in discussing the probability of selection bias. Thirdly, we cannot be certain of the extent to which those who responded understood the self-administered questionnaire or were motivated to give full and exact answers. Therefore, we cannot totally exclude information bias. If we assume that those who did not return the questionnaire were less knowledgeable of or cared less about research ethics, this could mean that non-responses biased the present result. However, this strengthens our argument that additional training is currently needed, as we can reasonably assume that participants’ knowledge of research ethics was probably overestimated.

## Conclusion

This study documented that only 40.4% of respondents had been trained in research ethics. The majority of them were not aware of the contents of international and national applicable ethical regulations of research involving human subjects. Consequently, few of those who had already implemented research projects reported having respected the autonomy of their research participants or submitting their research project to a competent REC. Most of respondents were aware of their role as future or potential researchers and/or REC members in the protection of research participants. From these results, we recommend that research ethics training should be included in the curriculum and obligatory for all students and residents before they start the end of course research projects.

## Additional file

Additional file 1: Data collection tool (Questionnaire). (DOCX 20 kb)

## Abbreviations

FMBS: Faculty of Medicine and Biomedical Sciences
REC: Research Ethics Committee
