# Supplementary material for: Biomedical research ethics in Cameroon: a survey to assess training needs of medical residents and students
Source: BMC Med Educ. 2019 Jan 3;19:5. doi: 10.1186/s12909-018-1431-8 (PMC6318839; doi:10.1186/s12909-018-1431-8)
Supplement: Supplementary file 1 — Data collection tool (Questionnaire). (DOCX 20 kb) [file 12909_2018_1431_MOESM1_ESM.docx]

Biomedical Research Ethics in Cameroon:A survey to assess training Needs of Medical Residents and Students

Questionnaire

| N° | Question | Response |
| --- | --- | --- |
|  | Date | _______________________________ |
|  | Respondent code | _______________________________ |
|  | Level of studies | 1. Medical student ; b) medical resident |
|  | For medical student, level? | 1. Level five ; b) level six |
|  | If medical resident, speciality | _______________________________ |
|  | Have you ever received any training in research ethics? | 1. Yes; b) no |
|  | If the answer to question 6 is yes, specify the training type | 1. Internet based, b) workshop, c) during internship, d) in academic milieu, e) others |
|  | If the answer to question 6 is yes, what is your level of satisfaction with the training received? | a)very satisfied, b) moderately satisfied, c) less satisfied d) not satisfied. |
|  | If the answer to question 6 is yes, are you willing to receive more training on the topic? | a)Yes; b) no |
|  | If the answer to question 6 is no, would you want to receive such training? | a)Yes; b) no |
|  | Do you have research ethics as one of the courses in your training program? | a)Yes; b) no |
|  | If the answer to question 11 is yes, list the various topics taught | ____________________________________________________________________________________________________________________________________________________________________________________________________________________________________________________________________________________________________________________________________________________________________________________________________________________________________________________________________________________________________________ |
|  | Have you ever been a PI or Co-PI in biomedical research involving human subjects? | a)Yes; b) no |
|  | If you answer in question 13 is “yes”, was the project submitted to IRB for ethical evaluation? | a)Yes; b) no |
|  | If you answer in question 13 is “yes”, did the project use informed consent to enrol subjects? | a)Yes; b) no |

In the table below, tick “I” if you are simple aware of the existence of regulation, “L” if you are aware of the content of the regulation or “N” if you have never heard of the regulation

| N° | I | L | N | Regulatory texts |
| --- | --- | --- | --- | --- |
|  |  |  |  | Nuremberge code |
|  |  |  |  | Declaration of Helsiki |
|  |  |  |  | International ethical guidelines for biomedical research involving human subjects |
|  |  |  |  | Declaration global medical association on ethical consideration with regards to health data base |
|  |  |  |  | Operational guidelines for ethics committees in charge of the evaluation of biomedical research |
|  |  |  |  | Belmont report |
|  |  |  |  | International conference on harmonisation (ICH) of guidelines for Good clinical practice (GCP) |
|  |  |  |  | 1991 international guidelines for ethical review of epidemiological studies |
|  |  |  |  | Universal declaration of human rights |
|  |  |  |  | Hippocrates oaths |
|  |  |  |  | Deontology code for medical practices in Cameroon |
|  |  |  |  | Ministerial order for the creation organisation and functioning of ethics committee of research involving human subject in Cameroon |
|  |  |  |  | Others precise__________________________________________________ |

In the table below, select the order of responsibility that you attribute to each research actor vis-a-vis the protection of research participants

| Actor | Level of responsibility | | | |
| --- | --- | --- | --- | --- |
|  | First person in charge | 2^nd^person in charge | 3^rd^person in charge | Does not have any responsibility at all |
| Investigator |  |  |  |  |
| Sponsor |  |  |  |  |
| Ethics committee |  |  |  |  |
| Research participants |  |  |  |  |
| Medical doctor who is caring for the patient out-of-research |  |  |  |  |
| The community |  |  |  |  |
| Other, precise_________ |  |  |  |  |

Please indicate the level of importance of the following research ethics training subjects. Use “1” =very important, “2” =averagely important, “3”=less important, “4” =not important

|  |  | Research regulation in Cameroon |
| --- | --- | --- |
|  |  | International research regulation |
|  |  | Fundamental principle of ethics: what are these principles,?what are their significance?, how can we interpret them? |
|  |  | Evaluation of social value of a research protocol |
|  |  | Evaluation of the scientific integrity of a research protocol |
|  |  | Equity in the selection of research participants |
|  |  | Evaluation of the risk/benefit ratio |
|  |  | Procedure of individual informed consent |
|  |  | Procedure of community informed consent |
|  |  | The notion of respect of research participants |
|  |  | Ethics committee: role, organisation, independence, authority, mandate, and responsibility |
|  |  | Level of accord between the local health needs and the objective of the project |
|  |  | Ethical issues for research sponsored by foreign bodies |
|  |  | Ethical issues in multicentre studies |
|  |  | Ethical issues in vulnerable population |
|  |  | Ethical issues in interventional studies |
|  |  | Ethical issues in research conducted on biological substances |
|  |  | Ethical principle in research involving archived data collected from research subjects |
|  |  | Ethical specificity of qualitative studies |
|  |  | The notion of conflict of interest |
|  |  | The nation of influence and coercion on research participants |
|  |  | The role and the responsibility of researchers for the protection of research participants |
|  |  | Compensation and repair of research damage to participants |
|  |  | Publication and author’s rights |
|  |  | Role and responsibility of sponsor in research |
